# A case-control study of reaction time deficits in a 3D virtual reality in patients with Post-COVID syndrome

**DOI:** 10.1038/s41598-024-76827-7

**Published:** 2024-11-08

**Authors:** Moritz Güttes, Marianna Lucio, Adam Skornia, Eva Rühl, Fritz Steußloff, Julia Zott, Christian Mardin, Wolfgang Mehringer, Marion Ganslmayer, Georg Michelson, Bettina Hohberger

**Affiliations:** 1 Department of Ophthalmology, Universitätsklinikum Erlangen, Friedrich-Alexander-Universität Erlangen- Nürnberg, Erlangen, Germany; 2 Research Unit Analytical BioGeoChemistry, Helmholtz Zentrum München, Neuherberg, Germany; 3 Department Artificial Intelligence in Biomedical Engineering (AIBE), Machine Learning and Data Analytics Lab (MaD Lab), Friedrich-Alexander-Universität Erlangen-Nürnberg (FAU), Erlangen, Germany; 4 Department of Internal Medicine 1, Universitätsklinikum Erlangen, Friedrich-Alexander-Universität Erlangen-Nürnberg, Erlangen, Germany

**Keywords:** Immunological disorders, Physical examination, Eye manifestations, Neurological manifestations, Pain, Disability

## Abstract

Following the Coronavirus disease 2019 (COVID-19) pandemic, a large number of people continue to report Post-COVID symptoms (PCS). A wide variety of symptoms are described, including fatigue, post-exertional malaise and cognitive impairment. However, adequate objective diagnostic tests for PCS are not yet available. Since the neurotropism of SARS-CoV-2 could be a possible factor for cognitive impairment, the aim of this study was to clarify whether visual reaction time (RT) in a stereoscopic setting can be a marker in PCS diagnostics. The Virtual-Reality-Oculomotor-Test-System (VR-OTS) was used testing binocular vision in 9 gaze directions via stereoscopic stimuli displayed in a virtual reality (VR)-environment (disparity: 275″, 550″, 1100″) in 179 individuals: 130 patients with PCS and 49 healthy controls. The results from the generalized linear models indicated that both group membership (PCS vs. control) and covariates (age and sex) yielded statistically significant different RT across the models. Accounting for the effect of covariates a statistically significant difference of RT was observed between patients with PCS and controls (disparity 275″ p-value = 0.001; 550″ p-value = 0.001; 1100″ p-value = 0.003). Patients with PCS performed worse in RT in all gaze directions, respectively. Adjusting for the influence of covariates, correct responses (CR) differed significantly between patients with PCS and controls (disparity 275″ p-value < 0.001; 550″ p-value = 0.003; 1100″ p-value = 0.019). Statistically significant effects of covariates on RT were observed for sex (disparity 275″ p-value = 0.047; 550″ p-value = 0.012; 1100″ p-value = 0.005) and age (disparity 275″ p-value < 0.001; 550″ p-value < 0.001; 1100″ p-value < 0.001). However, regarding covariates, no significant effects were found for CR, except for age at disparity 275″ (p-value = 0.035). The present data suggested that the mentioned variables uniquely contributed to explain the variation of the response variable (RT, CR). RT and CR detecting 3D-stimuli in a virtual 3D- environment might offer novel functional diagnostic approaches in PCS.

## Introduction

Coronavirus disease 2019 (COVID-19), a disorder caused by the severe acute respiratory syndrome coronavirus type 2 (SARS-CoV-2) became a global pandemic on March 11, 2020 ^1^. According to the WHO over 762 million confirmed cases of COVID-19, including over 6.8 million deaths were known worldwide until April 2023 ^2^.

Patients were not only suffering from acute COVID-19 symptoms, yet patients report of ongoing symptoms (Post-COVID syndrome, PCS). According to the WHO, “Post COVID-19 condition occurs in individuals with a history of probable or confirmed SARS CoV-2 infection, usually 3 months from the onset of COVID-19 with symptoms and that last for at least 2 months and cannot be explained by an alternative diagnosis”^3,4^. A global estimated prevalence of 12.7% ^5^ (ranging from 7.5 to 43% ^6,7^) in the general population after COVID-19 is estimated. Systematic reviews show a substantial prevalence of PCS^7,8^, yet data are still highly heterogeneous^8^. A wide range of PCS symptoms have been reported: in general, fatigue (including post-exertional malaise (PEM)), cardiac (e.g. tachycardia, postural orthostatic tachycardia syndrome, POTS), neurological (e.g. brain fog, sleep disorders), respiratory, gastrointestinal symptoms, and musculoskeletal pain^7–12^. Recent data indicated that 3 to less than 6 months after acute COVID-19, the most commonly reported symptoms were fatigue (including PEM), dyspnea, sleep disorder, and loss of concentration reported at rates of 32%, 25%, 24%, and 22% respectively^8^. At a follow-up of 6 to less than 9 months the most common symptoms were PEM, fatigue, sleep disorder, and dyspnea reported at rates of 45%, 36%, 29%, and 25% respectively^8^.

The exact pathogenesis is still elusive. Apparently, PCS seems to be a complex and multifactorial syndrome encompassing several subtypes (e.g. viral, autoimmune, vascular, and others)^13–16^. The neurotropism of SARS-CoV-2 may be a contributing factor to the neurological impairments^17,18^. There is evidence that cognitive impairment can occur in patients with all levels of initial COVID-19 severity^19^. Several diagnostic tests, addressing visual tasks, were suggested as possible assessment tools for brain health in patients with PCS^20–22^, and other brain related disorders^23–25^. Recent data showed a link between cognitive impairment and visual reaction time (RT) in 2-dimensional (2D) settings of patients with PCS^21,22^. Findings suggest that cognitive demand in a 3-dimensional (3D) virtual reality seems to differ in comparison to a 2D virtual reality^26^. Considering the neurotropism of SARS-CoV-2 ^17,18^, eye movement alterations in PCS^20^, and the correlation between cognitive impairment and RT in PCS^21,22^, it can be hypothesized that performance in 3-dimensional (3D) vision is reduced in patients with PCS. The aim of this study was to investigate whether RT and correct responses (CR) in a stereoscopic setting can be a distinguishing marker for PCS diagnostics.

## Materials and methods

### Study population

For a cross-sectional study 179 participants were recruited at the Department of Ophthalmology, Universitätsklinikum Erlangen, Friedrich-Alexander-Universität Erlangen-Nürnberg: 130 patients with PCS and 49 healthy controls. Patients were assigned to the PCS group in accordance with the German Post-Covid guideline^27^. Exclusion criteria were age below 18 years, pre-existing ocular disorders, pre-existing systemic disorders with ocular involvement, and an uncorrected visual acuity of the worse eye worse than 0.1 (LogMAR). The study has been reviewed and approved by the ethics committee of the Friedrich-Alexander-Universität Erlangen-Nürnberg (295_20 B) and was performed in accordance with the tenets of the Declaration of Helsinki. Informed written consent was achieved from each participant prior to enrollment.

### Virtual-reality (VR)-oculomotor-test-system (VR-OTS)

**Fig. 1:**
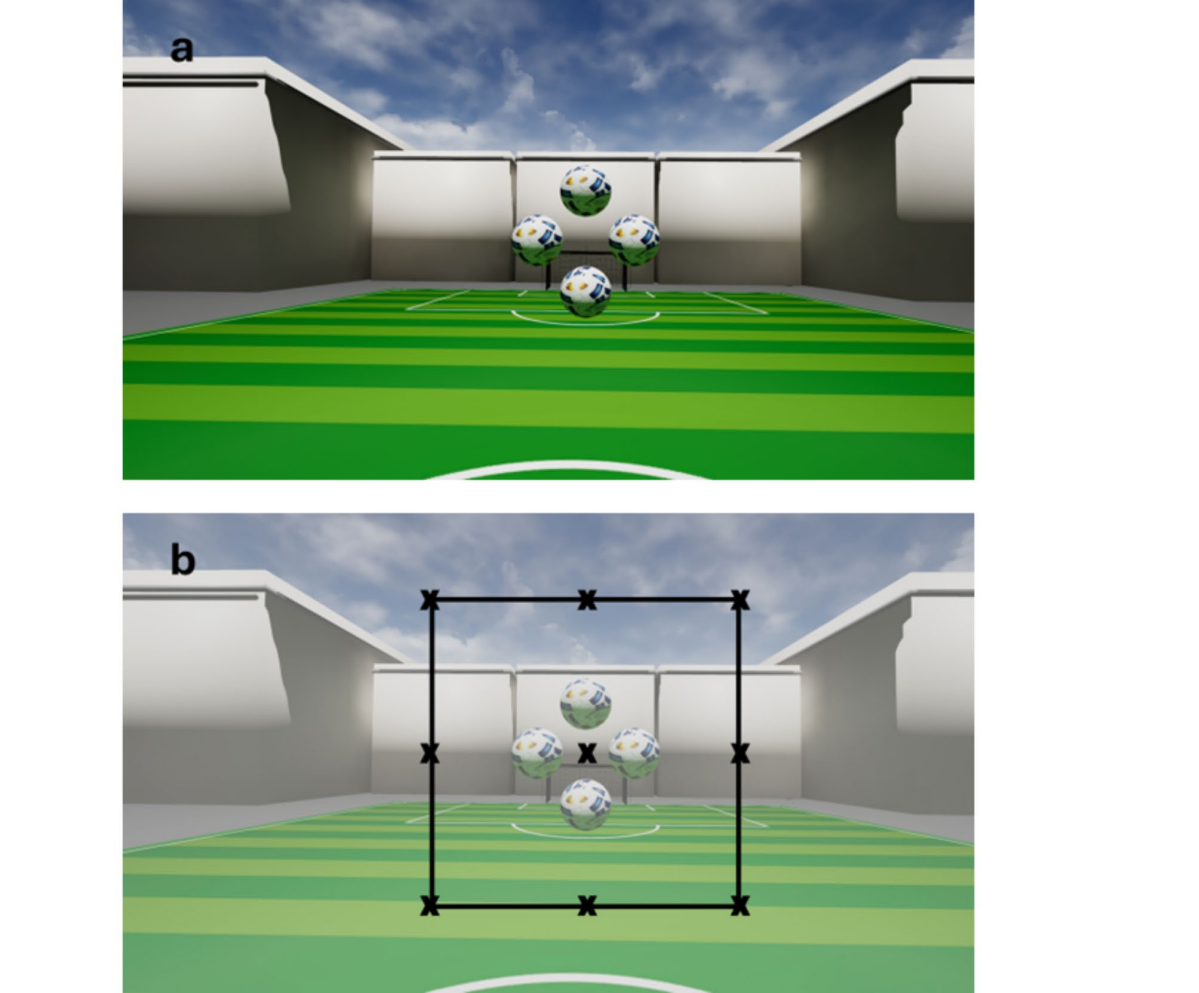
Virtual reality environment of the VR-OTS: (**a**) A sample image of the environment seen by the test person in the VR-OTs is shown. The environment has a stereoscopic impression of depth. The stimuli (four footballs) are floating in front of the user in a rhomboid arrangement in a stadium-like setting; (**b**) The different stimulus testing positions (gaze directions) are shown schematically. The black cross in the middle of the balls represents the center of this rhomboid arrangement. Nine different directions of gaze were defined in the user’s field of view. Each of the 9 crosses represents a possible test position of the stimuli. The example shown here shows the central position.

The Virtual-Reality-Oculomotor-Test-System (VR-OTS) is a virtual environment constructed to test 3D-vision/stereoscopic performance/depth-perception. VR-OTS is a Medical Device Regulation (MDR)-compliant class 1 medical device. The methodology was described in detail previously^28^. Shortly, as all four balls were the same size the following issues were addressed: linear perspective was solved by adding a random variation to each ball. Cue overlay of contours was canceled out by avoiding overlaps in the scene. Cue distribution of highlights and shadows was avoided by not casting shadows and using one light source that was far away. Aerial perspective was not rendered in the scene and motion parallax was avoided by attaching the stimulus to the user’s head rotation. Only binocular cues were effective. Monocular depth cues were eliminated, so the participant needed stereopsis to fulfill the task^28^. The task was presented with VR goggles and was based on binocular cues. The test procedure had to be carried out without the use of glasses or contact lenses. The VR environment showed a stadium-like setting (Fig. 1a): the stimuli (four footballs) were floating in front of the user in a rhomboid arrangement. The stimuli were shown in 9 gaze directions. At the start of the test, one of the balls appeared closer to the user. To create the illusion of one ball appearing closer than the others, a defined horizontal disparity difference was employed relative to the other balls. Three levels of difficulties were established using varying levels of this disparity: 250, 550, 1100 arc-seconds (arcsec, ″). The patients were asked to identify the closest ball of all and consequently to press the corresponding arrow key on a keyboard. The time required to select the correct stereoscopic stimulus is referred to as RT [ms]. CR was defined as percentage of correct responses [decimal]. Each of the three disparities was tested randomly three times at 9 different gaze directions, resulting in 81 inputs per test run. Test time was about 1 min/run (about 5 min/test, including introduction to the test set-up). As none of the participants had any previous experience with the test system, three test runs were done, yet the first two were not scored and used for the patient’s familiarization with the system. The third test run was used for statistical analysis.

### VR Headset

In this study, the HTC Vive Pro Eye (HTC Corporation, Taoyuan, Taiwan) was used to create a VR environment. The headset features two 3.5″ Organic-Light-Emitting-Diodes (OLED) displays, each with a resolution of 1440 × 1600 pixels and a refresh rate of 90 Hz. The manufacturer specifies a field of view (FoV) of 110◦. To compensate for variations in the user’s eye relief, the distance between the lenses can be adjusted in a range of 60–73 mm. In addition, the headset is equipped with a built-in Tobii eye tracking system (Tobii AB, Danderyd, Sweden), operating at a sampling rate of 120 Hz, thus achieving an accuracy of 0.5–1.1◦ within a 20◦ FoV. The movements of the headset are tracked in 3D space using HTC’s base stations. Our goal was to use purely stereoscopic visual stimuli that differ in their level of difficulty. For stereoscopic visual stimuli, the level of difficulty can be quantified by disparity differences. The minimum difference in disparities in our stereoscopic visual stimuli is 275″ per pixel^28^. In order to create different levels of difficulty, only a multiple of the value 275″ was allowed. Three difficulty levels were used (from highest difficulty to lowest difficulty: 275″, 550″, 1100″). By manipulating disparity differences, stimuli were created that would challenge participants’ perceptual abilities and allow for a precise measurement of their performance in virtual 3D vision.

### VR environment

The VR environment was designed as follows: the distance of each ball from the center was virtually 25 cm, either vertically or horizontally, and the four balls were defined as Ball-Up, Ball-Down, Ball-Left, and Ball-Right. These definitions were later used to identify, which ball the user was looking at. Nine different directions of gaze were defined within the user’s field of view (Fig. 1b), namely the 8 peripheral (down, lower right, right, upper right, up, upper left, left and lower left) and the central position. It was ensured that the user had to shift his gaze to focus on different stimulus locations. The VR environment followed the user’s head rotation and position so that the relative position of the stimuli to the user was always maintained and couldn’t be changed by turning the head.

### Statistical analysis

Variables age, visual acuity, and mean time since COVID-19 were presented before modelling using descriptive measures of center (mean) and variability (standard deviation). A series of generalized Linear Models (GLM) was employed to assess differences between controls and PCS patients, with age and sex included as covariates. This modeling approach, also known as ANCOVA, allowed us to account for the effects of age and sex while assessing the relationships between the primary variables of interest. Specifically, the GLMs were used to examine differences between PCS and control groups across various CR and RT measures, adjusting for these covariates. Separate GLMs were constructed for each independent variable to ensure precise evaluation. Moreover, we ran GLM models on data stratified by Control and PCS groups, with sex as the primary independent variable and age as a covariate. The response variables were CR and RT, with RT calculated as median RTs per each disparity, and gaze direction. The CR were calculated as percentages in decimal of correct inputs in relation to all inputs per each disparity, and gaze direction. Three levels of difficulty, disparities (275″, 550″, 1100″), were randomly tested three times each in nine different viewing directions (8 peripheral and one central position). To ensure robustness, p-values underwent Sidak adjustment. Estimates were calculated with their corresponding confidence interval (95% CIs) to reflect the precision of the results. Age-specific differences describe the estimated effect of age on the outcome, and whether this effect is statistically significant (p-value). Sex-specific differences describe the estimated effect of sex the outcome, and whether this effect is statistically significant (p-value). The two groups (control and PCS) had unequal sample sizes, therefore the LS-Means were calculated in order to provide more robust and accurate comparisons. The LS-Means are descriptive values (means) adjusted for the factors presented in the model (age and sex). Therefore, LS-Means provide a more robust estimate of the effect. Moreover, they are particularly useful in unbalanced designs or when comparing groups with unequal sample sizes. Additionally, Type III Sum of Squares ANOVA were computed to measure the unique contribution of each variable within the models.

To assess the overall significance of the models, F-Tests were conducted for each GLM. The F-Test evaluates whether the model, including the covariates and independent variables, explains a significant portion of the variability in the dependent variables. A significant F-Test indicates that the model as a whole is statistically significant. This was particularly important for confirming that the model appropriately captured the relationship between the primary variables of interest, while adjusting for age and sex. An ANOVA test was performed within each group (PCS and control) for 3D-performance for each disparity and gaze direction. This allowed us to evaluate whether any specific gaze direction was impaired. The dependent variables were CR and median RT, with gaze direction as factor. All the elaborations were done using the SAS version 9.4 (SAS Institute Inc., Cary, NC, USA) and Rstudio ggplot, dplyr and lm packages (Integrated Development for R. RStudio, PBC, Boston, MA).

## Results

### Demographic data

Of the 179 participants recruited 91 were female and 88 male, with mean age of 35.63 ± 11 years and with a mean uncorrected visual acuity of the worse eye of 0.02 ± 0.04 (LogMAR). The PCS group (130 patients, 66 female, 64 male, mean age 36.91 ± 11 years) had a mean uncorrected visual acuity of the worse eye of 0.02 ± 0.04 (LogMAR). The control group (49 healthy controls, 25 female, 24 male, mean age 32.24 ± 12 years) had a mean uncorrected visual acuity of the worse eye of 0.03 ± 0.04 (LogMAR). Mean time between acute COVID-19 and study participation was 442.49 ± 215 days for the PCS group. Self-reported PCS symptoms were fatigue (including PEM, 92%), reduced concentration (88%), headaches (75%), arrythmia (73%), muscle pain (68%), and POTS (63%).

### Overall performance of RT and CR

**Fig. 2:**
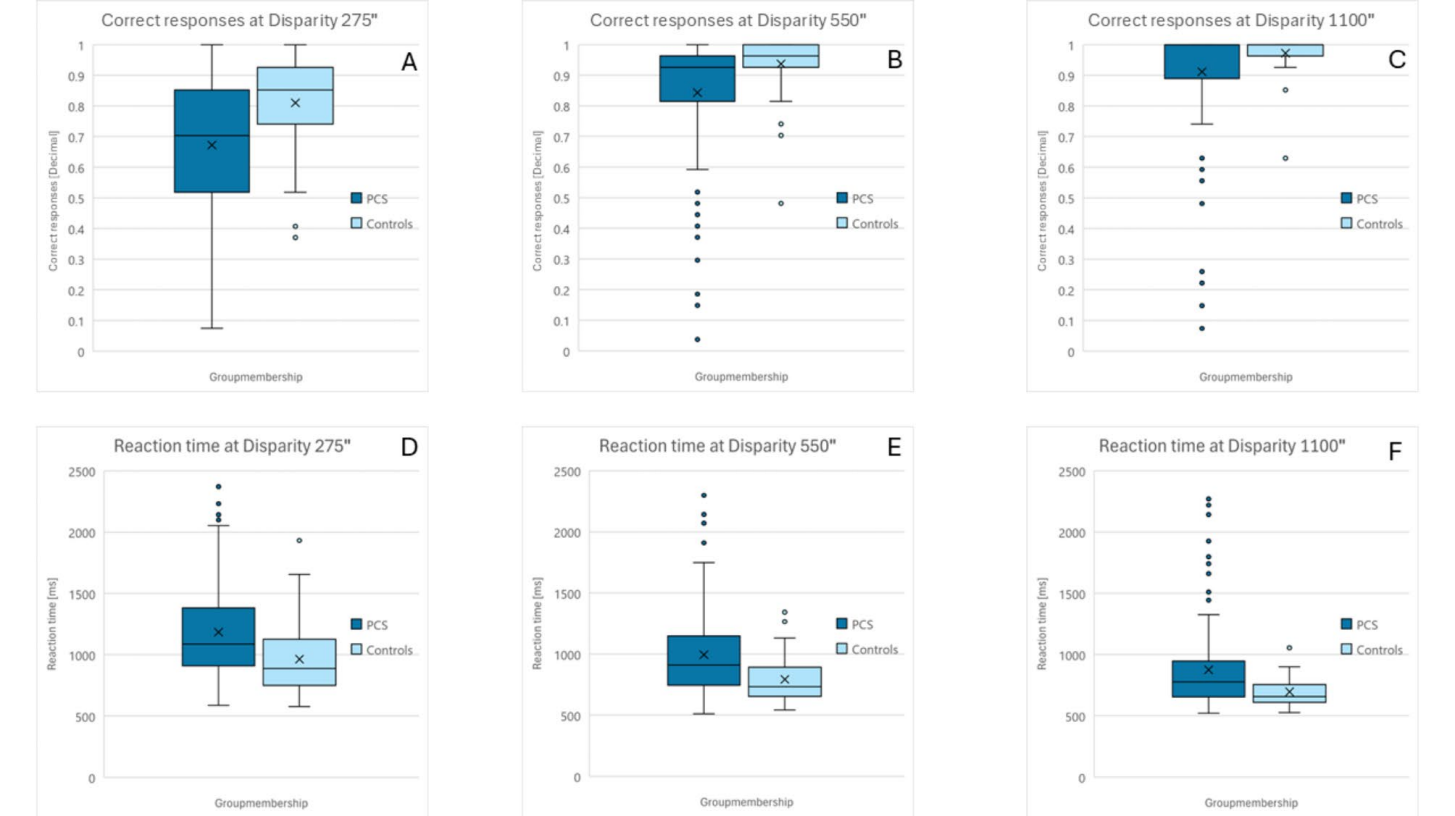
Boxplots of CR (**A**–**C**) and RT (**D**–**F**) in patients with PCS and controls across each disparity (275″, 550″, 1100″). Boxplots of LS-Means of CR [decimal] and RT [ms], adjusted for covariates. An ANCOVA test for group membership was done. Group membership shows statistically significant differences for CR (disparity 275″ p-value < 0.001, 550″ p-value = 0.003, 1100″ p-value = 0.019) and RT (disparity 275″ p-value = 0.001, 550″ p-value = 0.001, 1100″ p-value = 0.003) at all difficulties (disparities). Decreasing CR and increasing RT can be observed for increased difficulty (lower disparity) at all levels for both groups. (CR = correct responses; RT = reaction time; decimal = percentage in decimal; ms = milliseconds; PCS = Post-COVID syndrome; ″=arc-seconds; LS-Means = least square means)

**Fig. 3:**
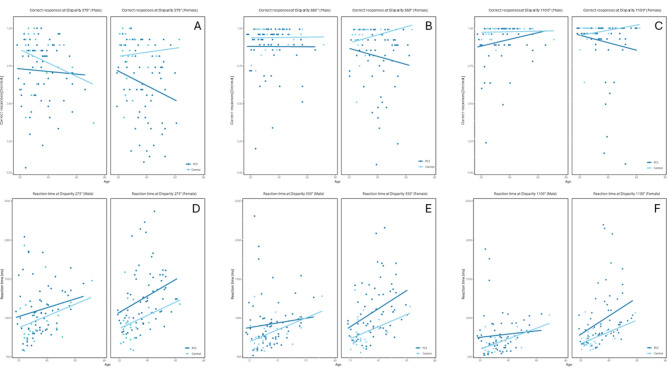
Scatterplots of CR (**A**–**C**) and RT (**D**–**F**) in patients with PCS and controls considering sex and age for each disparity (275″, 550″, 1100″). Scatterplots of LS-Means of CR [decimal] and RT [ms] are shown. The LS-Means adjust for potential differences between the groups concerning the covariates. Separate plots for males and females display the scored values on the y-axis and the participant’s age on the x-axis. A trend analysis, indicated by the solid line, was conducted. ANCOVA tests were performed for age and sex. Age showed to have statistically significant impact on RT (disparity 275″ p-value < 0.001, 550″ p-value < 0.001, 1100″ p-value < 0.001) at all difficulties (disparities), but for CR just at the hardest difficulty (disparity 275″ p-value = 0.035). Increasing age showed a trend to increase RT and decrease CR. Sex showed statistically significant impact on RT (disparity 275″ p-value = 0.047, 550″ p-value = 0.012, and 1100″ p-value = 0.005) and no statistically significant impact on CR for all disparities. Men exhibit lower RT compared to women. The PCS group’s trendline generally lies below that of the control group, indicating lower CR values across all age levels. Notable exceptions include CR at 275″ for males older than 60 years and RT at 550″ for males older than 60 years, as well as at 1100″ for males older than 55 years. (CR = correct responses; RT = reaction time; decimal = percentage in decimal; ms = milliseconds; PCS = Post-COVID syndrome; ″=arc-seconds; LS-Means = least square means)

In terms of overall performance differences in relation to group membership, PCS group showed statistically significant worse (higher) RT than control group in all disparity levels (Table 1), disparity 275″ (p-value = 0.001), 550″ (p-value = 0.001), 1100″ (p-value = 0.003) (Fig. 2D−F). The LS-Means of RT in the PCS group were higher across all difficulty levels (Table 1). It can also be observed that the RT increased with decreasing disparity in both groups (Fig. 3).

In terms of overall performance in relation to group membership, the CR showed statistically significant worse (lower) values of the PCS group than control group in all difficulty levels (Table 1), disparity 275″ (p-value = 0.001), 550″ (p-value = 0.003), 1100″ (p-value = 0.019) (Fig. 2A–C). The LS-Means of CR in the PCS group were lower across all difficulty levels (Table 1). It can also be observed that the CR in both collectives decreases with decreasing disparity (Fig. 3).

**Table 1:** Comparison of overall CR and RT per disparity between PCS and control groups. Output values of the GLMs models, which compare overall RT [ms] and CR [decimal] between control and PCS group at different disparity levels. It includes the estimated effect of age and sex specific differences on the response variable. Confidence intervals (95% CL) are included to show the range within which the true LS-Mean is expected to fall with 95% confidence. The table includes the relative p-values of the Type III Sum of square for each source of variation (group, age and sex); CR=correct responses; RT=reaction time; decimal=percentage in decimal; ms=milliseconds; PCS=Post-COVID syndrome; ″=arc-seconds; CL=confidence interval level; LS-Means=least square means; SE=standard errors.

Variables	Group	LS-Mean ± SE	95% CI	Group (*p*-value)	Age-specific Differences	Age (*p*-value)	Sex-specific Differences	Sex (*p*-value)
CR 275″	PCS	0.676 ± 0.0186	0.634–0.719	0.001	− 0.003	0.035	0.054	0.089
	Control	0.801 ± 0.0305	0.732–0.869					
CR 550″	PCS	0.844 ± 0.0157	0.809–0.88	0.003	− 0.001	0.459	0.046	0.067
	Control	0.935 ± 0.0256	0.877–0.993					
CR 1100″	PCS	0.911 ± 0.0134	0.881–0.942	0.019	0.0001	0.893	0.009	0.688
	Control	0.973 ± 0.022	0.923–1.022					
RT 275″	PCS	1173 ± 29.2	1107–1239	0.001	8.45	< 0.001	− 98.656	0.047
	Control	990 ± 47.7	883–1098					
RT 550″	PCS	985 ± 26.2	926–1044	0.001	7.778	< 0.001	− 112.49	0.012
	Control	818 ± 42.8	721–914					
RT 1100″	PCS	866 ± 25.1	809–922	0.003	7.16	< 0.001	− 120.008	0.005
	Control	719 ± 41	626–811					

### Sub-analysis of RT and CR according to gaze directions

A sub-analysis was conducted to compare RT and CR between PCS and control group across various individual gaze directions, in which the stimuli were presented (Tables 2 and 3).

In terms of performance in individual gaze direction the PCS group consistently showed increased RT across all disparities and gaze directions, with many of these differences reaching statistical significance (Table 2). At disparity 275″, five out of nine gaze directions showed a statistically significant difference (Table 2). At disparity 550″ and 1100″ all nine gaze directions showed a statistically significant difference (Table 2).

Similarly, for CR, patients with PCS consistently demonstrated lower CR across all disparities and gaze directions, with numerous differences achieving to be statistically significant (Table 3). At disparity 275″ six out of nine gaze directions, at disparity 550″ four out of nine gaze directions, and at disparity 1100″ three out of nine showed a statistically significant difference (Table 3).

No statistically significant differences (p-values > 0.5, provided by separate ANOVAs for PCS and control) were found between gaze directions for RT and CR across different disparities.

**Table 2:** Comparison of RT per gaze direction between PCS and control groups. Output values of the GLMs models, which compare RT [ms] per each gaze direction between control and PCS group at different disparity levels. It includes the estimated effect of age and sex specific differences on the response variable. Confidence intervals (95% CL) are included to show the range within which the true LS-Mean is expected to fall with 95% confidence. The table includes the relative p-values of the Type III Sum of square for each source of variation (group, age and sex); RT=reaction time; ms=milliseconds; PCS=Post-COVID syndrome; ″=arc-seconds; CL=confidence interval level; LS-Means=least square means; SE=standard errors.

Variables	Gaze Direction	Group	LS-Mean ± SE	95% CI	Group (*p*-value)	Age-specific Differences	Age (*p*-value)	Sex-specific Differences	Sex (*p*-value)
RT 275″	Down	PCS	1199 ± 37	1125–1273	0.044	7.863	0.009	85.31	0.178
		Control	1054 ± 60	935–1172					
	Lower right	PCS	1145 ± 38	1070–1219	0.079	6.559	0.02	32.683	0.602
		Control	1022 ± 58	908–1136					
	Right	PCS	1163 ± 38	1088–1238	0.546	9.82	0.001	29.13	0.646
		Control	1120 ± 58	1005–1235					
	Upper right	PCS	1181 ± 37	1108–1253	0.102	10.025	< 0.001	4.474	0.943
		Control	1066 ± 59	949–1182					
	Up	PCS	1177 ± 39	1100–1254	0.388	13.977	< 0.001	14.658	0.822
		Control	1115 ± 59	998–1233					
	Upper left	PCS	1232 ± 35	1162–1302	0.004	8.25	0.004	232.7	< 0.001
		Control	1030 ± 58	916–1144					
	Left	PCS	1255 ± 37	1182–1329	< 0.001	9.721	0.001	49.18	0.428
		Control	966 ± 59	850–1082					
	Lower left	PCS	1188 ± 37	1114–1261	0.016	10.507	< 0.001	107.783	0.087
		Control	1017 ± 59	899–1134					
	Central	PCS	1151 ± 36	1081–1222	< 0.001	3.786	0.164	166.272	0.006
		Control	876 ± 56	765–988					
RT 550″	Down	PCS	1002 ± 29	944–1059	0.002	6.81	0.003	135.717	0.006
		Control	824 ± 47	732–916					
	Lower right	PCS	1002 ± 28	946–1058	0.002	8.776	< 0.001	91.975	0.057
		Control	827 ± 46	737–917					
	Right	PCS	1027 ± 32	964–1089	0.003	8.645	< 0.001	47.712	0.372
		Control	848 ± 50	748–947					
	Upper right	PCS	1003 ± 31	941–1065	0.004	4.24	0.074	104.701	0.048
		Control	832 ± 49	734–929					
	Up	PCS	1036 ± 35	967–1105	0.017	6.954	0.01	88.822	0.135
		Control	874 ± 57	762–987					
	Upper left	PCS	1011 ± 32	947–1075	0.044	8.416	0.001	106.5	0.052
		Control	886 ± 52	784–988					
	Left	PCS	997 ± 31	947–1075	0.011	10.353	< 0.001	111.059	0.036
		Control	843 ± 50	784–988					
	Lower left	PCS	1014 ± 31	947–1075	0.003	9.905	< 0.0001	129.474	0.015
		Control	830 ± 51	784–988					
	Central	PCS	939 ± 28	884–995	0.013	8.183	< 0.001	137.533	0.004
		Control	805 ± 45	715–894					
RT 1100″	Down	PCS	891 ± 30	884–995	0.002	5.378	0.019	71.68	0.155
		Control	714 ± 48	715–894					
	Lower right	PCS	839 ± 23	884–995	0.002	4.392	0.013	100.007	0.011
		Control	704 ± 37	715–894					
	Right	PCS	886 ± 27	884–995	0.005	5.615	0.007	69.508	0.132
		Control	740 ± 44	715–894					
	Upper right	PCS	898 ± 28	844–952	0.004	5.994	0.005	139.82	0.003
		Control	742 ± 44	655–830					
	Up	PCS	877 ± 25	828–925	0.006	8.419	< 0.001	122.434	0.004
		Control	746 ± 40	667–824					
	Upper left	PCS	908 ± 28	828–925	< 0.001	7.152	0.001	114.842	0.018
		Control	708 ± 46	667–824					
	Left	PCS	907 ± 30	848–967	0.002	9.41	< 0.001	103.904	0.043
		Control	728 ± 49	631–824					
	Lower left	PCS	851 ± 26	801–902	0.031	7.628	< 0.001	81.761	0.061
		Control	745 ± 41	664–827					
	Central	PCS	848 ± 27	795–901	0.006	7.145	0.001	118.479	0.01
		Control	702 ± 44	615–789					

**Table 3:** Comparison of CR per gaze direction between PCS and control groups. Output values of the GLMs models, which compare CR [decimal] per each gaze direction between control and PCS group at different disparity levels. It includes the estimated effect of age and sex specific differences on the response variable. Confidence intervals (95% CL) are included to show the range within which the true LS-Mean is expected to fall with 95% confidence. The table includes the relative p-values of the Type III Sum of square for each source of variation (group, age and sex); CR=correct responses; decimal=percentage in decimal; PCS=Post-COVID syndrome; ″=arc-seconds; CL=confidence interval level; LS-Means=least square means; SE=standard errors.

Variables	Gaze direction	Group	LS-Mean ± SE	95% CI	Group (*p*-value)	Age-specific Differences	Age (*p*-value)	Sex-specific Differences	Sex (*p*-value)
CR 275″	Down	PCS	0.71 ± 0.026	0.659–0.76	0.083	− 0.005	0.006	0.018	0.675
		Control	0.795 ± 0.042	0.713–0.878					
	Lower right	PCS	0.658 ± 0.028	0.602–0.714	0.006	− 0.001	0.58	− 0.071	0.134
		Control	0.81 ± 0.046	0.719–0.902					
	Right	PCS	0.628 ± 0.028	0.572–0.684	0.009	− 0.002	0.306	− 0.067	0.166
		Control	0.773 ± 0.047	0.681–0.865					
	Upper right	PCS	0.697 ± 0.029	0.638–0.755	0.231	− 0.005	0.033	− 0.078	0.118
		Control	0.765 ± 0.048	0.67–0.86					
	Up	PCS	0.625 ± 0.031	0.565–0.686	0.001	0.0005	0.831	− 0.118	0.024
		Control	0.817 ± 0.05	0.719–0.916					
	Upper left	PCS	0.676 ± 0.026	0.624–0.729	0.134	− 0.004	0.05	− 0.045	0.318
		Control	0.753 ± 0.043	0.668–0.839					
	Left	PCS	0.644 ± 0.027	0.591–0.697	0.002	− 0.003	0.145	0.018	0.693
		Control	0.804 ± 0.044	0.717–0.891					
	Lower left	PCS	0.683 ± 0.026	0.632–0.735	0.017	− 0.003	0.138	− 0.055	0.227
		Control	0.805 ± 0.043	0.721–0.889					
	Central	PCS	0.727 ± 0.028	0.672–0.782	0.033	− 0.004	0.053	− 0.077	0.105
		Control	0.843 ± 0.046	0.753–0.933					
CR 550″	Down	PCS	0.871 ± 0.021	0.83–0.911	0.209	− 0.001	0.575	0.013	0.704
		Control	0.921 ± 0.034	0.854–0.988					
	Lower right	PCS	0.856 ± 0.02	0.816–0.896	0.052	− 0.002	0.324	− 0.039	0.265
		Control	0.933 ± 0.033	0.867–0.999					
	Right	PCS	0.816 ± 0.023	0.77–0.862	0.012	− 0.001	0.662	− 0.068	0.084
		Control	0.929 ± 0.038	0.854–1.004					
	Upper right	PCS	0.788 ± 0.025	0.739–0.837	0.005	− 0.001	0.624	− 0.09	0.032
		Control	0.922 ± 0.04	0.842–1.002					
	Up	PCS	0.797 ± 0.021	0.755–0.838	< 0.001	− 0.001	0.968	− 0.11	0.003
		Control	0.946 ± 0.035	0.878–1.014					
	Upper left	PCS	0.837 ± 0.024	0.79–0.884	0.128	0.001	0.544	− 0.035	0.386
		Control	0.908 ± 0.039	0.831–0.985					
	Left	PCS	0.852 ± 0.022	0.809–0.895	0.136	− 0.003	0.097	− 0.038	0.308
		Control	0.915 ± 0.036	0.845–0.986					
	Lower left	PCS	0.868 ± 0.02	0.829–0.908	0.058	− 0.001	0.484	− 0.039	0.25
		Control	0.942 ± 0.033	0.877–1.006					
	Central	PCS	0.871 ± 0.019	0.833–0.909	0.026	− 0.001	0.356	− 0.035	0.285
		Control	0.954 ± 0.031	0.892–1.016					
CR 1100″	Down	PCS	0.925 ± 0.014	0.897–0.953	0.02	0.001	0.319	0.016	0.506
		Control	0.99 ± 0.023	0.944–1.035					
	Lower right	PCS	0.912 ± 0.017	0.878–0.947	0.058	− 0.001	0.529	0.013	0.66
		Control	0.976 ± 0.028	0.92–1.032					
	Right	PCS	0.914 ± 0.017	0.88–0.949	0.195	− 0.0002	0.857	− 0.032	0.278
		Control	0.958 ± 0.028	0.902–1.014					
	Upper right	PCS	0.891 ± 0.019	0.853–0.929	0.046	− 0.0002	0.841	− 0.048	0.143
		Control	0.965 ± 0.031	0.903–1.027					
	Up	PCS	0.891 ± 0.018	0.855–0.926	0.035	− 0.0004	0.79	0.006	0.849
		Control	0.964 ± 0.029	0.906–1.022					
	Upper left	PCS	0.903 ± 0.018	0.868–0.939	0.167	0.00002	0.985	− 0.006	0.847
		Control	0.952 ± 0.03	0.894–1.01					
	Left	PCS	0.896 ± 0.018	0.86–0.932	0.082	-0.0004	0.782	0.009	0.76
		Control	0.957 ± 0.03	0.899–1.016					
	Lower left	PCS	0.923 ± 0.016	0.892–0.955	0.062	0.001	0.55	0.009	0.734
		Control	0.982 ± 0.026	0.93–1.033					
	Central	PCS	0.904 ± 0.018	0.868–0.94	0.176	− 0.002	0.177	− 0.021	0.492
		Control	0.951 ± 0.03	0.893–1.01					

### Analysis of the covariates (age, sex) on RT and CR

We analyzed the effects of the covariates age and sex on RT and CR in the total cohort (Table 1). Statistically significant effects of age on RT were observed across all disparities, 275” (p-value < 0.001), 550” (p-value < 0.001), and 1100” (p-value < 0.001), indicating an increasing trend in RT with age (refer to Fig. 3D-F). Men generally exhibited lower RTs than women, with significant differences at disparities 275” (p-value = 0.047), 550” (p-value = 0.012), and 1100” (p-value = 0.005). Regarding CR, age showed a statistically significant impact only at the disparity of 275” (p-value = 0.035), with a negative trend in CR values as age increased (Fig. 3A). No significant age effects on CR were observed at disparities of 550” (p-value = 0.459) and 1100” (p-value = 0.893). Additionally, no statistically significant differences were found for the effect of sex on CR across all disparities (275” p-value = 0.089, 550” p-value = 0.067, and 1100” p-value = 0.688).

In addition, we performed a sub-analysis of the effect of sex on the variables (RT and CR) for patients with PCS and controls, respectively (Table 4). RT of female patients with PCS was significantly increased across all disparities (275” p-value = 0.03, 550” p-value = 0.015, and 1100” p-value = 0.009) compared to male patients with PCS. Contrary, RT was not statistically significantly different between the sexes in controls (disparity 275” p-value = 0.991, 550” p-value = 0.548, and 1100” p-value = 0.29). Regarding CR, sex showed no statistically significant difference for RT or CR in the PCS or control group, except for CR at disparity 275” of PCS (p-value = 0.044, Table 4).

**Table 4:** Analysis of the effect of sex on RT and CR separated by group membership. Output values of the GLMs models on stratified data by group (PCS and control), with sex as the primary independent variable and age as a covariate. It includes the estimated effect of age and sex specific differences on the response variable. Confidence intervals (95% CL) are included to show the range within which the true LS-Mean is expected to fall with 95% confidence. We analyzed the effect of sex on the variables (RT [ms] and CR [decimal]) for PCS and controls each. The table includes the relative p-values of the Type III Sum of square for each source of variation (sex, age); CR=correct responses; RT=reaction time; decimal=percentage in decimal; ms=milliseconds; PCS=Post-COVID syndrome; ″=arc-seconds; CL=confidence interval level; LS-Means=least square means; SE=standard errors.

Variables	Sex	LS-Mean ± SE	95% CI	Age-specific differences	Age (*p*-value)	Sex-specific differences	Sex (*p*-value)
CR 275″ (subgroup Control)	Male	0.8 ± 0.034	0.732–0.868	-0.002	0.248	0.02	0.676
	Female	0.82 ± 0.033	0.754–0.886				
CR 275″ (subgroup PCS)	Male	0.713 ± 0.028	0.657–0.769	-0.003	0.127	− 0.081	0.044
	Female	0.632 ± 0.028	0.576–0.688				
CR 550″ (subgroup Control)	Male	0.937 ± 0.021	0.894–0.979	0.001	0.411	0.001	0.974
	Female	0.938 ± 0.021	0.896–0.98				
CR 550″ (subgroup PCS)	Male	0.874 ± 0.025	0.825–0.923	-0.001	0.37	− 0.062	0.078
	Female	0.812 ± 0.025	0.763–0.861				
CR 1100″ (subgroup Control)	Male	0.976 ± 0.013	0.95–1.002	0.001	0.357	− 0.008	0.648
	Female	0.968 ± 0.013	0.943–0.993				
CR 1100″ (subgroup PCS)	Male	0.915 ± 0.022	0.872–0.959	-0.0001	0.93	− 0.007	0.809
	Female	0.908 ± 0.022	0.865–0.951				
RT 275″ (subgroup Control)	Male	963 ± 56.9	848–1077	7.79	0.03	0.8203	0.82
	Female	964 ± 55.7	851–1076				
RT 275″ (subgroup PCS)	Male	1116 ± 43.7	1030–1202	8.101	0.005	135.298	0.03
	Female	1251 ± 43.3	1165–1337				
RT 550″ (subgroup Control)	Male	777 ± 37	702–851	7.655	0.001	31.807	0.548
	Female	809 ± 36.3	736–882				
RT 550″ (subgroup PCS)	Male	922 ± 41.3	841–1004	7.296	0.008	144.079	0.015
	Female	1066 ± 41	985–1148				
RT 1100″ (subgroup Control)	Male	678 ± 22.5	633–724	6.885	< 0.0001	34.313	0.29
	Female	713 ± 22	668–757				
T 1100″ (subgroup PCS)	Male	798 ± 40.7	718–879	6.715	0.013	153.1	0.009
	Female	951 ± 40.4	871–1031				

## Discussion

With the emergence of the SARS-CoV-2 pandemic, a symptom complex called PCS is increasingly coming into focus alongside various acute and extensive sequelae. A broad variety of symptoms are reported, which appear to affect a wide range of organ systems^7–12^, including partly severely debilitating neurological and cognitive symptoms^19,29,30^. PCS can result in a massive decrease of patients’ quality of life: patients can feel moderate up to severe impairment performing household tasks or general work^31^, some aren’t able to work^6,32^ or even need intense care in all aspects of life^33,34^. Thus, PCS can have a serious impact on people’s autonomy, mental health, their social life, and their ability to go back to work^35^. This results in a significant economic impact for the patients themselves and society, respectively^6,35^. Right now, an objective diagnosis or objectively standardized diagnostic criteria are not yet available and are needed for regular clinical all-day life^36^. SARS-CoV-2 is known to show a neurotropism^17,18^. Considering eye movement alterations in patients with PCS^20^ and a correlation between cognitive impairment and RT in patients with PCS in a 2D-environment^21,22^, it can be hypothesized that performance in 3-dimensional (3D) vision is reduced in patients with PCS. This study investigated the RT during virtual-3D-environment testing in patients with PCS and found a worsening compared to controls. Visual tasks demanding stereoscopic capability were performed using a virtual reality setting. The PCS patients performed significantly worse in all difficulties regarding CR and RT. Our sub-analysis revealed no significant discrepancy in any gaze direction to be particularly impaired among the observed difficulties, therefore it can be argued that the worse performance of PCS seems to be homogeneously distributed across all gaze directions. Looking at the different performance per level of difficulty for both groups poorer performance was found in terms of CR and RT with increasing difficulty achieved through decreasing disparities.

Different testing approaches for measuring visuals tasks in patients after COVID-19 were described^20–22,28^. A video-oculography study design showed eye movement alterations after symptomatic COVID-19 in comparison to controls^20^. The combination of a questionnaire of self-reported Long-COVID symptoms and a self-administered visual detection task yielded that the parameters ‘bad cognition’, ‘poor physical condition’, ‘learning difficulties’, and ‘visual impairment’ were the best predictors for a prolonged RT in their 2D-visual tasks^21^. Yet, these data were collected without comparison to a control group. An oculomotor, vestibular, RT test system with eye tracking showed altered optokinetic nystagmus and saccades in patients after COVID-19 compared to normative data^22^.

All these methods used a 2D-environement, contrary to the VR-OTS method, using a virtual-3D-environment. The VR-OTS method has already been used in a proof-of-concept study^28^. The method is based on previous works from Paulus et al.^37^, and Schoemann et al.^38^. A stereoscopic task uses different disparities between the test objects. The task was presented with VR goggles and was based solely on binocular cues. For stereoscopic visual stimuli, the level of difficulty was quantified by disparity differences. The minimum difference in disparities in our stereoscopic visual stimuli were 275″ per pixel^28^. By manipulating disparity differences, stimuli that would challenge participants’ perceptual abilities were created.

Virtual 3D testing was described in several brain associated diseases like Parkinson’s disease^23,24^, Alzheimer’s disease^23^, mild traumatic brain injuries^25^, and depression^30^ previously. Ba et al.^24^ used a 3D active shutter system and found patients with Parkinson’s disease to have worse stereopsis, longer visual response times, and eye movement alterations, which correlated positively with motor function and negatively with cognitive status. Kara et al.^25^ used the VR-OTS in a similar manner than this study used, in patients with concussion. They showed patients with mild and moderate traumatic brain injury to have impaired stereopsis. The response time and error rate differences were statistically significant. Paulus et al.^37^ were using a 3D-TV with a static and dynamic stereo test, one consisting of 4 soccer balls and found no significantly better performance of soccer players against no soccer player in their stereopsis tests, even though they performed better in a monocular simple choice test regarding RT. They also found a significant increase in RT with decreasing disparity differences. Effects on stereopsis were also investigated in non-disease related contexts such as sports^37^ and aging^39^.

The results of the present study confirm the previous data of a proof-of-concept study^28^. Both variables, RT and CR, were significantly impaired in patients with PCS compared to controls (Table 1). As the parameter CR represents the correct responses, RT and CR do not affect each other. Both parameters showed a similar impairment in any gaze direction. Other studies also showed a worse performance in visual tasks in PCS collectives^21,22,40^. Santoyo-Mora et al. showed the need of a higher visual sensitivity and processing speed after severe COVID-19 ^40^. Significant differences in RT for choosing the correct stereoscopic stimulus were found ranging from 147 ms, 167 ms to 183 ms for RT from easiest to hardest difficulty.

For the effect of age on CR the PCS group’s trendline consistently remained in a lower position compared to the controls, indicating an effect on all levels of age (Fig. 3). For the effect of age on RT statistically significant results were found. A trend that RT is increasing with an increased age was observed. This could be attributed to the described effect of aging on processing speed^41–43^. Studies suggested a link between visual impairment and cognitive decline in middle ages and older people^44–46^. Crivelli et al.^10^ also showed a rise in cognitive dysfunction after COVID-19 with increasing age.

In our data set male and female participants performed significantly differently regarding RT (Table 1). The RT of female PCS patients was increased compared to male patients at all levels of difficulty, yet not in controls (Table 4). Regarding the sex effect for CR, no sex effect was observed in either PCS or controls, except for PCS at disparity 275″ (Table 4). These findings could support the trend, in which women exhibited cognitive impairment more often than male after COVID-19 ^36^.

The observed impaired RT and CR in patients with PCS could be attributed to different factors: neuronal perception time, motoric reaction and maximum speed (including the muscles of the eye), neuronal recording time and processing time could contribute singularly or combined to a decreasing stereoscopic performance. The findings of the present study might be associated with prolonged saccade latencies, impaired saccade accuracy, increased corrective movements and fixations. More fixations lead to longer processing times as the brain has to integrate more visual data. In addition, impaired smooth pursuit movements might lead to fragmented and less efficient visual tracking. Further studies could address the singular factors for an enhanced understanding of the pathomechanistic alterations.

The study is not without limitations. Visual acuity was required to not be worse than 0.1 (logMAR) (without correction), as the test set-up did not allow wearing of glasses during the VR-OTS test. In addition, the study design was cross-sectional. It would be of interest, if long-term data could confirm an improvement or worsening of the PCS symptoms over a period of time. As the VR-OTS test duration is even short (about 5 min) and easy to handle, the VR-OTS seems to be suitable for patients with fatigue or PEM.

## Conclusion

The data of the present study showed that a stereoscopic impairment of RT and CR might be a novel diagnostic marker in PCS diagnostics.

## Data Availability

The datasets are available from the corresponding author upon reasonable request.
